# Pharmacokinetics of Teicoplanin in a Patient with Coronavirus Disease 2019 Receiving Veno-venous Extracorporeal Membrane Oxygenation

**DOI:** 10.2478/jccm-2022-0021

**Published:** 2022-11-12

**Authors:** Nobuhisa Hirayu, Atsuo Nakamura, Toshio Morita, Osamu Takasu

**Affiliations:** 1 Department of Emergency and Critical Care Medicine, Kurume University School of Medicine, Kurume, Fukuoka, Japan

**Keywords:** coronavirus, drug-resistant bacteria, intensive care management, teicoplanin, veno-venous extracorporeal membrane oxygenation, volume of distribution

## Abstract

**Introduction:**

Patients with severe coronavirus disease 2019 (COVID-19) receiving ventilation or pulmonary support via veno-venous extracorporeal membrane oxygenation (VV-ECMO) can be infected with drug-resistant bacteria. When introducing VV-ECMO, the changes in serum antibiotic concentration should be considered due to an increased volume of distribution (Vd). However, no pharmacokinetic study has assessed teicoplanin (TEIC) treatment in patients with COVID-19 receiving VV-ECMO.

**Case presentation:**

A 71-year-old man diagnosed with COVID-19 visited a primary hospital. His oxygenation conditions worsened despite treatment with favipiravir and methylprednisolone as well as oxygen therapy. After his transfer to our center, tracheal intubation and steroid pulse therapy were initiated. Seven days after admission, VV-ECMO was performed. TEIC was administered for secondary bacterial infection. The serum TEIC concentration remained within the therapeutic range, indicating that VV-ECMO did not significantly affect TEIC pharmacokinetics. VV-ECMO was discontinued 17 days after admission. However, he developed multi-organ disorder and died 42 days after admission.

**Conclusion:**

As TEIC prevents viral invasion, it may be used with ECMO in patients with COVID-19 requiring ventilation; however, the altered pharmacokinetics of TEIC, such as increased Vd, should be considered. Therefore, TEIC pharmacokinetics in VV-ECMO should be assessed in future studies with an appropriate number of patients.

## Introduction

The novel coronavirus infection that started in Wuhan (Hubei Province, China) in December 2019 is a respiratory infection caused by severe acute respiratory syndrome coronavirus-2. Few patients with coronavirus disease 2019 (COVID-19) respond to existing antiviral agents. However, currently, there are no targeted therapies against COVID-19 [1]; only symptomatic treatments are available. Patients with severe COVID-19 require invasive artificial-ventilation therapy or further pulmonary support through veno-venous extracorporeal membrane oxygenation (VV-ECMO) [2]. Patients receiving such an aggressive mechanical support therapy are at a high risk of developing complications such as ventilator-associated pneumonia (VAP) [3] and catheter-related bloodstream infection (CRBSI) [4]. The risk of infection by drug-resistant bacteria such as methicillin-resistant *Staphylococcus aureus* (MRSA) should also be considered [5]. In VV-ECMO therapy, drug concentration changes due to an increased volume of distribution (Vd) should be considered [6]. However, there have only been a few pharmacokinetic studies on teicoplanin (TEIC), a glycopeptide antibiotic with anti-MRSA activity [7,8].

Herein, we describe the pharmacokinetics of TEIC under VV-ECMO therapy in a patient with respiratory failure caused by COVID-19 with a secondary bacterial infection.

## Case Presentation

A 71-year-old man with fever and malaise as initial symptoms underwent PCR testing 4 days after symptom onset and was diagnosed with COVID-19 on the following day. He was prescribed favipiravir and methylprednisolone (125 mg/day) and was administered oxygen at 7 L/min via a face mask. Owing to gradual worsening of oxygenation, the patient was transferred to an emergency center 7 days after the onset.

The patient’s medical history included hypertension, chronic obstructive pulmonary disease, type 2 diabetes mellitus, total gastrectomy, and splenectomy. His vital signs were stable at admission, except percutaneous oxygen saturation of 95% at a low rate of 15 L/ min oxygen via a reservoir mask. In breath sound assessment, fine crackles were found to extend in both lungs. Several laboratory findings including lactate dehydrogenase, ferritin, C-reactive protein, and D-dimer levels showed values beyond the normal range (Table 1). In contrast, the lymphocyte counts were below the normal range. Blood gas findings indicated hypoxemia.

**Table 1:** Blood test results at admission

Tests	Results
**Complete blood count**	
WBC	9,200/μL
Neut	81%
Ly	12.5%
Hb	15.3 g/dL
Hct	44.3%
Plt	217 × 103/μL
**Blood coagulation**	
PT	125%
APTT	33.2 s
Fibrinogen	535 mg/dL
FDP	5.7 μg/mL
D-dimer	1.9 μg/mL
AT III	98%
TAT	11.1 ng/mL
**Blood biochemistry**	
AST	86 U/L
ALT	34 U/L
LDH	833 U/L
TP	5.5 g/dL
Alb	2.7 g/dL
BUN	48 mg/dL
Cr	0.77 mg/dL
Na	139 mEq/L
K	3.9 mEq/L
Cl	104 mEq/L
CK	136 U/L
Ferritin	882.1 ng/dL
CRP	3.6 mg/dL
PCT	0.3 ng/mL
**Blood gas (oxygen mask with reservoir and oxygen flow set at 15 L/min)**	
pH	7.489
PaCO_2_	29.9 Torr
PaO_2_	62.3 Torr
HCO_3_	22.5 mEq/L
BE	0.7 mEq/L
SatO_2_	91.9%
Lac	1.2 mmol/L

WBC, white blood cell; Neut, neutrophil; Ly, lymphocyte; Hb, hemoglobin; Ht, hematocrit; Plt, platelet; PT, prothrombin time; APTT, activated partial thromboplastin time; FDP, fibrin/fibrinogen degradation products; AT, antithrombin activity; TAT, thrombin-antithrombin complex; AST, aspartate aminotransferase; ALT, alanine transaminase; LD, lactate dehydrogenase; TP, total protein; Alb, albumin; BUN, blood urea nitrogen; Cr, creatinine; Na, sodium; K, potassium; Cl, chlorine; CK, creatine kinase; CRP, c-reactive protein; PCT, procalcitonin; BE, base excess; Lac, lactate

On day 2 after admission, due to the worsening of oxygenation and reduced level of consciousness, mechanical ventilation via tracheal intubation was performed (FiO_2,_ 0.8 cmH_2_O; Positive End Expiratory Pressure (PEEP), 10 cmH_2_O) along with the initiation of steroid pulse therapy (1000 mg methylprednisolone for 3 days). The chest radiograph showed increased bilateral diffuse mixed consolidation and ground-glass opacity. His oxygenation gradually worsened, with fever, increased inflammatory response, and increased tracheal secretions. The SOFA score was 4 on admission but increased to 11, suggesting progression to organ failure. In addition, *Staphylococcus* species were detected in surveillance cultures. Ventilator settings were then changed to the following: FiO_2_, 1.0; P/F, 88 and PEEP, 12 cmH_2_O. Prominent hypoxemia was also observed. Seven days after admission, VV-ECMO was introduced (Murray Score: 2.5) (venous drainage through the right internal jugular vein with a 23-Fr cannula, venous reinfusion through the right femoral vein with a 19-Fr HLS cannula [Maquet Getinge, Rastatt, Germany], MERA NHP Exelung HPO-23WH-C [Senko Medical Instrument, Tokyo, Japan]). VAP and CRBSI were suspected due to an increased inflammatory response and decreased P/F ratio; thus, meropenem and TEIC were prescribed.

**Fig. 1:**
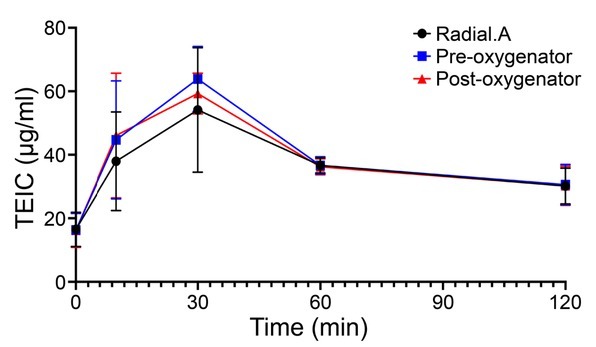
Time course of teicoplanin (TEIC) concentration in the blood after administering the maintenance dose of 600 mg TEIC every 24 h. Blood was collected from the radial artery (black) and pre-oxygenator site (blue) and post-oxygenator (red) site.

High-dose loading of TEIC was performed according to the dosing regimen (therapeutic administration regimen) of the intensive care unit [9]. TEIC (800 mg) was administered four times every 12 h, followed by a maintenance dose of 600 mg of TEIC every 24 h. The serum creatinine and albumin levels at the beginning of VV-ECMO were 0.65 mg/dL and 2.4 g/dL, respectively. Renal replacement therapy was not performed during VV-ECMO. On day 3 after initiating VV-ECMO (before the 5^th^ administration of TEIC), the serum TEIC concentration reached the target level (19.5 μg/mL). Serum TEIC concentrations were measured from different sites (radial artery, before and after oxygenation) at 0, 10, 30, 60, and 120 min after TEIC administration to determine the presence of adsorption by ECMO for three days from day five to day seven (during TEIC maintenance administration) (Figure 1).

The serum TEIC concentrations before administration of the maintenance dose were not significantly different among the three sites. The serum TEIC concentration peaked at 30 min post-administration. Although the concentration decreased over time, the serum TEIC level remained in the therapeutic range (15–25 mg/mL) without decreasing below the effective range. Even after the introduction of VV-ECMO, an elevated inflammatory response was observed, and the involvement of CRBSI and VAP was suspected. However, we determined that ECMO weaning was difficult until respiratory status improved and TEIC was continued. However, since MRSA was not identified from sputum culture and blood culture, TEIC was terminated after 14 days.

VV-ECMO and prone positioning therapy were effective, and the patient showed an improvement in oxygenation. VV-ECMO was discontinued 17 days after admission (11 days after the introduction of VV-ECMO). However, the patient persistently presented with intractable diarrhea caused by cytomegalovirus and died 42 days after admission due to multiple organ failure caused by VAP.

Written informed consent was obtained from the patient’s next of kin for the publication of this case report, with the preservation of the patient’s anonymity.

## Discussion

Our regimen successfully maintained the serum TEIC concentration after the initial high-dose loading and subsequent therapeutic drug dosing during VV-EC-MO. The concentration of a drug depends on several factors including its protein binding rate, Vd, and tissue penetration. Pharmacokinetics during ECMO requires the consideration of increased Vd caused by not only the performance status of patients (e.g., edema), but also by an increased circulating blood volume due to the ECMO circuit as well as sequestration of the drug to the ECMO circuit, cardiac output, and vascular hyperpermeability [6].

There have been a few pharmacokinetic studies on TEIC during ECMO [7,8]; however, none have been performed in patients with COVID-19 receiving VV-ECMO. TEIC is a water-soluble antibacterial agent with a remarkably high protein binding rate (90%) and Vd (0.85–1.0 L/kg) that is twice as high as that of vancomycin (an anti-MRSA agent). In cases of hypoalbuminemia, the protein binding rate of TEIC is altered; its free concentration and Vd are increased, whereas its total concentration is reduced [10,11]. Specifically, the protein binding rate of TEIC is reduced (58%) and its free concentration is increased (22%) in patients with hypoalbuminemia (median, 1.61 g/dL) [10].

TEIC is sequestered to a specific membrane during renal replacement therapy [12]. As the membrane oxygenator of the ECMO machine used in our patient contained a polypropylene membrane coated with a skin-like silicone layer, removal of TEIC by adsorption to a certain extent was a concern. However, drug monitoring in the patient showed that the serum TEIC concentrations with and without the use of the oxygenator during ECMO were not significantly different, thus suggesting that TEIC was not sequestered by the ECMO oxygenator membrane.

In patients with severe pneumonia receiving VV-ECMO, a therapy management strategy that focuses on the treatment of VAP and CRBSI along with MRSA coverage is vital. While therapeutic options with MRSA coverage include vancomycin and other anti-MRSA agents, TEIC results in a significantly lower risk of developing nephrotoxicity and red man syndrome when compared to vancomycin [13]. Interestingly, *in vitro* studies have shown that TEIC also prevents viral spread by inhibiting the activation of cathepsin L [14]. The COVID-19 pandemic is an ongoing global disaster, and the development of effective therapeutic agents is imperative. Therefore, we suggest that TEIC may be used as a therapy for bacterial and viral infections, as it may lower the risk of developing adverse events. Furthermore, it may be useful in addition to ECMO as a standard therapy to prevent viral invasion in patients with severe COVID-19 who require anti-MRSA treatment; however, further studies on this treatment will be needed.

## Conclusions

In summary, the serum levels of TEIC did not differ before and after the use of an oxygenator during V-V ECMO induction in our case. However, this study was limited because measurements were taken over only three days in one case.
